# Supplementary material for: Evaluation of an Oral Care Program to Improve the Oral Health of Home-Dwelling Older People
Source: Int J Environ Res Public Health. 2022 Jun 13;19(12):7251. doi: 10.3390/ijerph19127251 (PMC9223830; doi:10.3390/ijerph19127251)
Supplement: Supplementary file 1 [file ijerph-19-07251-s001.zip › ijerph-1729310-supplementary.pdf]

File S1 – questionnaires HCNs: MIDI and knowledge & attitude questionnaire – in Dutch/ English
File S2 – assessment older people, OHAT Dutch, oral (self) care questionnaire - in Dutch and English version
File S3 – Results MIDI questionnaire
File S4 – SQUIRE checklist

File S1 - MIDI questionnaire (in Dutch)
Over het Sûne Mûle Project
Er volgen een aantal stellingen. Geef aan in hoeverre je het eens bent met de stelling.

1. Het Sûne Mûle Project geeft helder aan wat er van jullie verwacht wordt
(1) helemaal mee oneens
(2) mee oneens
(3) noch mee oneens, noch mee eens
(4) mee eens
(5) helemaal mee eens

2. Het Sûne Mûle Project is gebaseerd op feitelijk juiste kennis
(1) helemaal mee oneens
(2) mee oneens
(3) noch mee oneens, noch mee eens
(4) mee eens
(5) helemaal mee eens
(6) weet ik niet

3. Het Sûne Mûle Project biedt alle informatie en materialen die nodig zijn om dit project tot een succes te maken
(1) helemaal mee oneens
(2) mee oneens
(3) noch mee oneens, noch mee eens
(4) mee eens
(5) helemaal mee eens

4. Het Sûne Mûle Project is te ingewikkeld voor mij
(1) helemaal mee oneens
(2) mee oneens
(3) noch mee oneens, noch mee eens
(4) mee eens
(5) helemaal mee eens
5. Het Sûne Mûle Project sluit goed aan bij hoe ik gewend ben om te werken.
(1) helemaal mee oneens
(2) mee oneens
(3) noch mee oneens, noch mee eens
(4) mee eens
(5) helemaal mee eens

6. Ik vind de effecten van het Sûne Mûle Project duidelijk zichtbaar
(1) helemaal mee oneens
(2) mee oneens
(3) noch mee oneens, noch mee eens
(4) mee eens
(5) helemaal mee eens

7. Ik vind het Sûne Mûle Project geschikt voor mijn cliënten
(1) helemaal mee oneens

(2) mee oneens
(3) noch mee oneens, noch mee eens
(4) mee eens
(5) helemaal mee eens

Over jou als thuiszorgmedewerker
Er volgen een aantal stellingen. Geef aan in hoeverre je het eens bent met de stelling.
8. Cliënten worden zich bewust van het belang van een goede mondverzorging door het Sûne Mûle Project
(1) helemaal mee oneens
(2) mee oneens
(3) noch mee oneens, noch mee eens
(4) mee eens
(5) helemaal mee eens

9. Het Sûne Mûle Project kost de thuiszorgmedewerkers veel tijd
(1) helemaal mee eens
(2) mee eens
(3) noch mee oneens, noch mee eens
(4) mee oneens
(5) helemaal mee oneens

10. Ik vind het belangrijk om met het Sûne Mûle Project de mondgezondheid van kwetsbare thuiswonende ouderen te verbeteren
(1) helemaal mee oneens
(2) mee oneens
(3) noch mee oneens, noch mee eens
(4) mee eens
(5) helemaal mee eens

11. Ik verwacht dat met het Sûne Mûle Project de mondgezondheid van mijn cliënten daadwerkelijk verbetert
(1) zeer zeker niet
(2) zeker niet
(3) misschien niet, misschien wel
(4) zeker wel
(5) zeer zeker wel

12. Ik vind het bij mijn functie horen om de dagelijkse mondverzorging van mijn cliënten uit te voeren en/ of hen daarbij te ondersteunen
(1) helemaal mee oneens
(2) mee oneens
(3) noch mee oneens, noch mee eens
(4) mee eens
(5) helemaal mee eens

13. Ik vind het bij mijn functie horen om bij mijn cliënten te signaleren hoe het is gesteld met hun mondverzorging/ mondgezondheid
(1) helemaal mee oneens

(2) mee oneens
(3) noch mee oneens, noch mee eens
(4) mee eens
(5) helemaal mee eens

14. Cliënten zullen over het algemeen tevreden zijn over het Sûne Mûle Project
(1) helemaal mee oneens
(2) mee oneens
(3) noch mee oneens, noch mee eens
(4) mee eens
(5) helemaal mee eens

15. Cliënten zullen over het algemeen meewerken aan het Sûne Mûle Project
(1) helemaal mee oneens
(2) mee oneens
(3) noch mee oneens, noch mee eens
(4) mee eens
(5) helemaal mee eens

16. Ik kan op voldoende hulp van mijn collega's rekenen mocht ik die nodig hebben in het Sûne Mûle Project
(1) helemaal mee oneens
(2) mee oneens
(3) noch mee oneens, noch mee eens
(4) mee eens
(5) helemaal mee eens

17. Hoe groot is volgens jou het deel collega's in jouw team die ook daadwerkelijk met het Sûne Mûle Project mee doen?
(1) geen enkele collega
(2) bijna geen enkele collega
(3) een minderheid
(4) de helft
(5) een meerderheid
(6) bijna alle collega's
(7) alle collega's

18. In hoeverre verwachten de personen die hieronder genoemd worden, dat je mee doet met het Sûne Mûle Project?

	zeer zeker niet	zeker niet	misschien niet, misschien wel	zeker wel	zeer zeker wel
De zorgverzekeraar	☐	☐	☐	☐	☐
Het Sûne Mûle Team	☐	☐	☐	☐	☐
De manager	☐	☐	☐	☐	☐
De organisatie	☐	☐	☐	☐	☐
Mijn collega's/ teamgenoten	☐	☐	☐	☐	☐

19. Als het gaat om het werken zoals dat in het Sûne Mûle Project wordt voorgesteld, hoeveel trek je je dan aan van de mening van de personen die hieronder worden genoemd?

	zeer weinig	weinig	niet weinig, niet veel	veel	zeer veel
De zorgverzekeraar	☐	☐	☐	☐	☐
Het Sûne Mûle Team	☐	☐	☐	☐	☐
De manager	☐	☐	☐	☐	☐
De organisatie	☐	☐	☐	☐	☐
Mijn collega's/ teamgenoten	☐	☐	☐	☐	☐

20. Indien je dat zou willen, denk je dat het je dan lukt om de dagelijkse mondverzorging van jouw cliënten uit te voeren en/of daarbij te ondersteunen?
(1) zeer zeker niet
(2) zeker niet
(3) misschien niet, misschien wel
(4) zeker wel
(5) zeer zeker wel

21. Indien je dat zou willen, denk je dat het je dan lukt om bij jouw cliënten te signaleren hoe het is gesteld met hun mondverzorging/ mondgezondheid?
(1)zeer zeker niet
(2) zeker niet
(3) misschien niet, misschien wel
(4) zeker wel
(5) zeer zeker wel

22. In hoeverre ben je op de hoogte van de inhoud van het Sûne Mûle Project?
(1) ik ken het Sûne Mûle Project niet
(2) ik ken het Sûne Mûle Project, maar ik weet nog niets van de inhoud
(3) ik ken het Sûne Mûle Project en heb een idee wat het project inhoudt
(4) ik ken het Sûne Mûle Project en weet wat het project inhoudt

Over de omgeving

23. Zijn in jouw organisatie/ team formeel afspraken vastgelegd door het management over het meedoen aan het Sûne Mûle Project (in beleidsplannen, werkplannen en dergelijke)?
(1) nee
(2) ja
(3) dat weet ik niet

24. Onze organisatie stelt mij voldoende tijd beschikbaar om de activiteiten van het Sûne Mûle Project in te passen in mijn dagelijks werk
(1) helemaal mee oneens
(2) mee oneens
(3) noch mee oneens, noch mee eens
(4) mee eens
(5) helemaal mee eens

25. In mijn team zijn één of meerdere personen aangewezen om de invoering van het Sûne Mûle Project te coördineren
(1) nee
(2) ja
(3) dat weet ik niet

26. Zijn er, behalve de invoering van het Sûne Mûle Project, andere veranderingen waarmee je momenteel of binnen afzienbare tijd mee te maken hebt (reorganisatie, fusie, bezuinigingen, personeelsverloop, andere vernieuwingen)?
(1) nee
(2) ja
(3) dat weet ik niet

27. Ik heb in mijn team makkelijk toegang tot informatie en materialen van het Sûne Mûle Project
(1) helemaal mee oneens
(2) mee oneens
(3) noch mee oneens, noch mee eens
(4) mee eens
(5) helemaal mee eens

28. In mijn team vindt regelmatig terugkoppeling plaats over de voortgang van het Sûne Mûle Project
(1) helemaal mee oneens
(2) mee oneens
(3) noch mee oneens, noch mee eens
(4) mee eens
(5) helemaal mee eens

29. Sûne Mûle Project sluit goed aan bij bestaande wetten, regels en protocollen/ richtlijnen
(1) helemaal mee oneens
(2) mee oneens
(3) noch mee oneens, noch mee eens
(4) mee eens
(5) helemaal mee eens

MIDI questionnaire (in English)

About the Oral Care Program
Statements are given below. Mark to what extend you agree with the statement.
1. The Oral Care Project is clear in what is expected from us
(1) completely agree
(2) agree
(3) nor agree, nor disagree
(4) disagree
(5) completely disagree

2. The Oral Care Project is based on actually true knowledge
(1) completely agree
(2) agree
(3) nor agree, nor disagree
(4) disagree
(5) completely disagree
(6) I don't know

3. The Oral Care Project provides all information and materials that are needed to make the project succeed
(1) completely agree
(2) agree
(3) nor agree, nor disagree
(4) disagree
(5) completely disagree

4. The Oral Care Project is too complicated for me
(1) completely agree
(2) agree
(3) nor agree, nor disagree
(4) disagree
(5) completely disagree

5. The Oral Care Project fits well in my usual way of working
(1) completely disagree
(2) disagree
(3) nor agree, nor disagree
(4) agree
(5) completely agree

6. I think the effects of the Oral Care Program are clearly visible
(1) completely disagree
(2) disagree
(3) nor agree, nor disagree
(4) agree
(5) completely agree

7. I think the Oral Care Project is suitable for my clients

(1) completely disagree
(2) disagree
(3) nor agree, nor disagree
(4) agree
(5) completely agree

About you as home care nurse
Statements are given below. Mark to what extend you agree with the statement.
8. Clients become aware of the importance of good oral hygiene through the Oral Care Program
(1) completely disagree
(2) disagree
(3) nor agree, nor disagree
(4) agree
(5) completely agree

9. The Oral Care Project takes a lot of time of home care nurses
(1) completely agree
(2) agree
(3) nor agree, nor disagree
(4) disagree
(5) completely disagree

10. I think it is important to improve the oral health of frail home dwelling older people, with the Oral Care Program
(1) completely disagree
(2) disagree
(3) nor agree, nor disagree
(4) agree
(5) completely agree

11. I think that the Oral Care Program really improves the oral health of my clients
(1) most certainly not
(2) certainly not
(3) maybe not, maybe it does
(4) certainly
(5) most certainly

12. I think it is part of my job to support or to do clients with daily oral care
(1) completely disagree
(2) disagree
(3) nor agree, nor disagree
(4) agree
(5) completely agree

13. I think it is part of my job to identify the status of my clients' oral care or oral health
(1) completely disagree
(2) disagree
(3) nor agree, nor disagree

(4) agree
(5) completely agree

14. Generally, clients will be content about the Oral Care Program
(1) completely disagree
(2) disagree
(3) nor agree, nor disagree
(4) agree
(5) completely agree

15. Overall, clients shall be willing to participate in the Oral Care Program
(1) completely disagree
(2) disagree
(3) nor agree, nor disagree
(4) agree
(5) completely agree

16. I can count on enough support of my colleagues if I may need it, in the Oral Care Program
(1) completely disagree
(2) disagree
(3) nor agree, nor disagree
(4) agree
(5) completely agree

17. How big is the proportion of colleagues in your team that will actually participate in the Oral Care Program, according to you?
(1) none of my colleagues
(2) almost none of my colleagues
(3) a minority of my colleagues
(4) half of my colleagues
(5) a majority of colleaguesy
(6) almost all my colleagues
(7) all my colleagues

18. To what extend do the persons mentioned below, expect you to participate in the Oral Care Program?

	definitely not	not	maybe not, maybe yes	yes	definitely yes
The health insurance company	☐	☐	☐	☐	☐
Team of dental hygienists	☐	☐	☐	☐	☐
The manager	☐	☐	☐	☐	☐
The home care organization	☐	☐	☐	☐	☐
My colleagues/ team members	☐	☐	☐	☐	☐

19. If it is about working as proposed in the Oral Care Program, how much do you care about the opinion of the persons mentioned below?

	Very little	little	not little, not much	much	very much
The health insurance company	☐	☐	☐	☐	☐
Team of dental hygienists	☐	☐	☐	☐	☐
The manager	☐	☐	☐	☐	☐
The home care organization	☐	☐	☐	☐	☐
My colleagues/ team members	☐	☐	☐	☐	☐

20. If you would be willing to, do you think you would be able to perform/ or to support with daily oral care of your clients?
(1) definitely not
(2) not
(3) maybe not, maybe yes
(4) yes
(5) definitely yes

21. If you would be willing to, do you think you would be able to identify the status of clients oral health or oral care?
(1) definitely not
(2) not
(3) maybe not, maybe yes
(4) yes
(5) definitely yes

22. To what content do you know the content of the Oral Care Program?
(1) I do not know the Oral Care Program
(2) I know the Oral Care Program, but I do not know anything about the content
(3) I know the Oral Care Program and I have an idea about the content
(4) I know the Oral Care Program and I do know the content

About the environment
23. Are there agreements documented by the management within your in organization/ team about participating in the Oral Care Project? (in policy, working plans etcetera)
(1) no
(2) yes
(3) I do not know

24. Our organization offers me enough time available to fit the activities of the Oral Care Program into my daily work.
(1) completely disagree
(2) disagree
(3) nor agree, nor disagree
(4) agree
(5) completely agree

25. In my team one or more persons are designated to coordinate the implementation of the Oral Care Program

(1) no
(2) yes
(3) I do not know

26. Are there any changes expected for you in the near future alongside the implementation of the Oral Care Project? (examples are reorganization, merges, retrenchments, staff turnover, other innovations)
(1) no
(2) yes
(3) I do not know

27. I have access to information and materials of the Oral Care Project easily
(1) completely disagree
(2) disagree
(3) nor agree, nor disagree
(4) agree
(5) completely agree
28. In my team we have regular feedback moments about the progress of the Oral Care Program
(1) completely disagree
(2) disagree
(3) nor agree, nor disagree
(4) agree
(5) completely agree

29. The Oral Care Project corresponds well with existing laws, regulations, protocols and guidelines
(1) completely disagree
(2) disagree
(3) nor agree, nor disagree
(4) agree
(5) completely agree

Knowledge and attitude questionnaire (Dutch) for HCNs
Vragenlijst voor thuiszorgmedewerkers over mondverzorging

Welkom bij deze vragenlijst. Je moet deze vragen zelf invullen en niet te lang nadenken over de vragen. Het invullen zou niet meer dan 5 -7 minuten van je tijd in beslag moeten nemen.

Als je het moeilijk vindt om een antwoord te kiezen, kies dan het antwoord dat het meest bij je past. Het is belangrijk voor de kwaliteit van het onderzoek dat zoveel mogelijk vragen zijn ingevuld.

1. Geslacht ☐ Man ☐ Vrouw

2. Je leeftijd: …………………..

3. Beroep: (Kruis je hoogst afgeronde opleiding aan)
   ☐ MBO Zorghulp (niveau 1)
   ☐ MBO Helpende Zorg en Welzijn (niveau 2)
   ☐ MBO Verzorgende (niveau 3)
   ☐ MBO verpleegkunde (niveau 4)
   ☐ HBO Verpleegkunde (niveau 5)
   ☐ Anders, namelijk………………………………………..

4. Hoe lang ben je werkzaam bij de thuiszorg? Aantal jaren ………………

Zowel eigen tanden en kiezen als een kunstgebit moeten schoongehouden en verzorgd worden om mondproblemen te voorkomen.<br>Onder *'mondverzorging'* verstaan we in deze vragenlijst alle dagelijkse mondverzorging om tanden, kiezen, implantaten, (gedeeltelijke) kunstgebitten, andere constructies en slijmvliezen en tandvlees schoon te houden. Een voorbeeld is tandenpoetsen.

5. Heb je in je opleiding (vraag 3) les gehad over mondverzorging bij cliënten?

   ☐ nee
   ☐ ja

6. Heb je een aanvullende cursus of nascholing gevolgd na je studie, over mondverzorging bij cliënten?
   ☐ Nee
   ☐ Ja, bij een eerdere werkgever
   ☐ Ja, bij mijn huidige werkgever
   ☐ Ja, bij beide/ meerdere werkgevers

7. Heb je op dit moment behoefte aan bij- en/of nascholing over mondverzorging voor cliënten?
   ☐ nee
   ☐ ja

8. In hoeverre vind je dat mondverzorging aandacht krijgt op je huidige werkplek? Kruis een rapportcijfer aan. Cijfer 1 is heel weinig aandacht, cijfer 10 is heel veel aandacht.

1	2	3	4	5	6	7	8	9	10
☐	☐	☐	☐	☐	☐	☐	☐	☐	☐

9. Over jouw werkplek: In hoeverre ben je het eens met de volgende uitspraken? (Kruis aan)

	Heel erg eens	Eens	Oneens	Helemaal oneens
A. Ik denk dat cliënten een gezonde mond belangrijk vinden	☐	☐	☐	☐
B. Onze cliënten vragen niet om ondersteuning bij mondverzorging	☐	☐	☐	☐
C. Ik vind het belangrijk dat ook de mond van onze clienten wordt verzorgd	☐	☐	☐	☐
D. Ik heb genoeg vaardigheden voor mondverzorging van cliënten	☐	☐	☐	☐
E. Ik heb genoeg tijd voor de mondverzorging van cliënten	☐	☐	☐	☐
F. Er zijn genoeg middelen/ materialen voor mondverzorging (tandenborstels etc.) beschikbaar	☐	☐	☐	☐
G. Ik vind dat mondverzorging ook onderdeel moet zijn van het individuele zorgplan van cliënten	☐	☐	☐	☐
H. Ik voel me onzeker om mondverzorging bij cliënten uit te voeren	☐	☐	☐	☐
I. Ik vind het vervelend om mondverzorging bij cliënten uit te voeren	☐	☐	☐	☐
J. Ik vind het moeilijk om mondverzorging bij cliënten uit te voeren	☐	☐	☐	☐

10. Welke van de bovenstaande uitspraken (vraag 9), vormt voor jou de grootste uitdaging, wat betreft de mondverzorging voor cliënten?

☐ 9 A (belang wat cliënt hecht)
☐ 9 B (cliënt vraagt niet om ondersteuning)
☐ 9 C (eigen belang)
☐ 9 d (vaardigheden)
☐ 9 E (tijd)
☐ 9 F (middelen)
☐ 9 G (zorgplan)
☐ 9 H (onzeker)
☐ 9 I (vervelend)
☐ 9 J (moeilijk)

11. Hoe vaak geef je uit eigen initiatief bij de cliënt aan dat de mondverzorging/ mondgezondheid van de cliënt aandacht nodig heeft?
 Nooit
 Af en toe, maar relatief zelden
 Enkele keren in de loop van de week
 Ongeveer elke keer als ik bij cliënten kom

12. Hoe vaak vragen cliënten aan jou, om hen te ondersteunen bij hun mondverzorging?
 Nooit
 Af en toe, maar relatief zelden
 Enkele keren in de loop van de week
 Ongeveer elke keer als ik bij cliënten kom

13. Trek je handschoenen aan bij het uitvoeren van mondverzorging bij cliënten?

 nooit soms altijd niet van toepassing (doe geen mondzorg)

14. Wie is volgens jou verantwoordelijk voor het signaleren van mondproblemen/ veranderingen in de mondverzorging van cliënten? (meerdere antwoorden mogelijk)
 Thuiszorgmedewerker
 Tandarts/ mondhygiënist
 De cliënt zelf
 Familie of mantelzorgers van de cliënt
 (Huis)arts/ POH
 Alle personen die hierboven worden genoemd
 Weet ik niet
 Anders, namelijk.....

15. Veel cliënten die thuiszorg ontvangen, gaan niet (meer) naar een tandarts of mondhygiënist. Kruis hieronder 2 redenen aan die volgens jou het meest vaak de oorzaak daarvoor zijn.

 Cliënten vinden het niet nodig (bijv. al lang een kunstgebit)
 Cliënten hebben geen klachten (bijv. geen pijn)
 Cliënten zijn niet meer mobiel/ geen vervoer
 Cliënten zijn te ziek
 Cliënten zeggen afspraken af
 Cliënten krijgen geen oproep meer
 Lange wachtlijsten/ geen tandarts of mondhygiënist beschikbaar
 Cliënten geven geen prioriteit aan mondzorg in verband met andere (gezondheids)problemen
 Cliënten vergeten afspraken
 Cliënten zijn bang voor de kosten

	Juist	Onjuist	Weet ik niet
1. Het verliezen van tanden of kiezen hoort bij ouder worden			
2. Medicijngebruik kan een nadelig effect hebben op of in de mond			
3. Het is voor oudere mensen voldoende om één keer per dag hun tanden te poetsen			
4. Mensen met een (gedeeltelijk) kunstgebit kunnen deze het best 's nachts inhouden			
5. Cliënten die een kunstgebit dragen hoeven alleen naar de tandarts/ mondhygiënist, wanneer ze een probleem hebben			
6. Zacht of vloeibaar voedsel is gezonder voor de mond van ouderen			
7. Een vies kunstgebit ziet er niet aantrekkelijk uit, maar dat kan geen mondaandoeningen veroorzaken			
8. Gewichtsverlies en een slechte mondgezondheid kunnen met elkaar te maken hebben			
9. Een slechte mondverzorging kan bijdragen aan het ontstaan van schimmelinfecties in de mond			
10. Een slechte mondgezondheid kan een negatieve invloed hebben op de kwaliteit van leven van cliënten			
11. Een slechte mondgezondheid kan de bloedsuikerspiegel van cliënten met diabetes ontregelen			
12. Bij cliënten met een droge mond ontstaan minder snel gaatjes (cariës) en tandvleesproblemen			
13. Een zachte tandenborstel is beter dan een harde om de natuurlijke (eigen) tanden en kiezen van een cliënt te poetsen			
14. Als cliënten vaker dan 7 x per dag zoete voedingsmiddelen eten en drinken, zijn hun tanden vatbaarder voor gaatjes (cariës)			
15. Wanneer tandvlees bloedt bij zachtjes poetsen, is het beter om te stoppen met poetsen			
16. Wanneer een thuiszorgmedewerker de mond van cliënten verzorgt of daarbij ondersteunt, moeten handschoenen worden gedragen			
17. Het dragen van een kunstgebit heeft geen invloed op de bacteriën die in de mond van de cliënt aanwezig zijn			
18. Het spoelen van de mond met water, is een goed alternatief voor het tandenpoetsen			

19. Als een kunstgebit ‘s nachts wordt gedragen/ ingehouden, kan het tandvlees onder het kunstgebit ontstoken raken			
20. Een droge mond wordt vaak veroorzaakt door medicijngebruik			
21. Een kunstgebit mag niet met tandpasta worden gepoetst			
22. Kosten voor tandheelkundige zorg moeten altijd door de cliënt zelf worden betaald of worden vergoed via de aanvullende tandverzekering van de cliënt			
23. Een slechte mondgezondheid kan een longontsteking veroorzaken			
24. Gaatjes (cariës) en tandvleesontsteking worden altijd veroorzaakt door bacteriën			
25. Als mensen uit hun mond ruiken, komt dat meestal door maag- of longproblemen			
26. Producten om kunstgebitten te reinigen (zoals een reinigingstablet) maken het kunstgebit schoon, zonder dat je het kunstgebit nog hoeft te poetsen			
27. Wanneer cliënten alleen maar sondevoeding krijgen, is mondverzorging niet meer nodig			
28. Implantaten in de mond kunnen het best met rust gelaten worden (bijvoorbeeld niet poetsen)			
29. Het gebruiken van een tandpasta met fluoride er in, is voor ouderen met natuurlijke (eigen) tanden en kiezen, belangrijk			

Knowledge and attitude questionnaire for HCNs

Welcome to this questionnaire. You need to complete these questions by yourself and do not think too long about the questions. Completing the questionnaire should not take more than 5-7 minutes.
If you find it difficult to choose an answer, choose the answer that matches mostly. It is important to the quality of the research project, to complete as much questions as possible.

1. Gender □ Male □ Female

2. Your age: ………………….

3. Profession: (Mark highest level of education)
   □ Care helper (level 1)
   □ Nurse Assistant/ aide (level 2)
   □ Certified nurse assistant (level 3)
   □ Licensed practical nurse (level 4)
   □ Registered Nurse/ Bachelor degree (level 5)
   □ different, ……………………………………….

4. How long do you work in formal home care nursing? Number of years……………….

Both natural teeth and dentures should be cleaned on a daily basis, to prevent oral problems. In this questionnaire, 'oral care' means all daily oral care to clean natural teeth, dental implants and (partial) dentures, gums and soft tissues in and around the mouth. An example is brushing teeth.

5. Was oral care for clients part of your vocational training (question 3)?

   □ no
   □ yes

6. Did you follow additional courses, after your vocational training, about oral care for clients?
   □ No
   □ Yes, at a former employer
   □ Yes, at my current employer
   □ Yes, at both/ multiple employers

7. Do you require additional education about oral care for clients, at this moment?
   □ no
   □ yes

8. In your opinion, to what extent oral care gets 'attention' in your current work area?
Mark a figure, figure 1 is very little attention, figure 10 is very much attention.

1	2	3	4	5	6	7	8	9	10
☐	☐	☐	☐	☐	☐	☐	☐	☐	☐

9. About your work area: to what extent do you agree with the following statements? (please mark)

	Completely agree	Agree	Disagree	Completely disagree
A. I think clients find a healthy mouth important	☐	☐	☐	☐
B. Our clients do not ask support for oral care	☐	☐	☐	☐
C. I think it is important to also care for our clients' mouth	☐	☐	☐	☐
D. I have enough skills to perform oral care for our clients	☐	☐	☐	☐
E. I have enough time to perform oral care for our clients	☐	☐	☐	☐
F. There are enough materials available to perform oral care (tooth brushes etcetera)	☐	☐	☐	☐
G. I think oral care should be part of the clients' general personal care plan	☐	☐	☐	☐
H. I feel insecure to perform oral care for clients	☐	☐	☐	☐
I. I think it is an unpleasant task to perform care for clients		☐	☐	☐ oral
J. I think it is difficult to perform oral care for clients	☐	☐	☐	☐

10. Which of above mentioned statements (question 9) is the greatest barrier to perform oral care for clients, to you?

☐ 9 A (importance of oral care to client)
☐ 9 B (client does not ask for support)
☐ 9 C (importance of oral care)
☐ 9 d (skills)
☐ 9 E (time)
☐ 9 F (materials for oral care)
☐ 9 G (personal care plan)
☐ 9 H (unsure)
☐ 9 I (unpleasant)
☐ 9 J (difficult)

11. How often do you start a conversation with clients, about the importance of oral care/ oral health?
☐ Never
☐ Occasionally
☐ A few times each week
☐ About every time I visit clients

12. How often do clients ask your support with oral care?
- ☐ Never
- ☐ Occasionally
- ☐ A few times each week
- ☐ About every time I visit clients

13. Do you wear gloves when you perform oral care in clients?

☐ never ☐ sometimes ☐ always ☐ not applicable (no oral care)

14. Who is responsible for noticing oral problems/ changes in oral health (care) of clients? Multiple answers possible
    - ☐ Home care nurse
    - ☐ Dentist/ dental hygienist
    - ☐ Client
    - ☐ Family or informal care givers of the client
    - ☐ General Doctor/ Nurse Practitioner
    - ☐ all persons stated above
    - ☐ I do not know
    - ☐ different…….

15. Many clients who make use of formal home care nursing, do not visit a dentist/ dental hygienist. What are to you, the 2 main reasons? Mark two reasons below.

    - ☐ Clients find it unnecessary (e.g. have dentures for a long time)
    - ☐ Clients have no complaints (e.g. no pain)
    - ☐ Clients have lack of transportation/ not mobile
    - ☐ Clients are too ill
    - ☐ Clients cancel appointments
    - ☐ Clients do not get reminders from dentists/ dental hygienists
    - ☐ Waiting lists/ no dentist or dental hygienist available
    - ☐ Clients have other priorities (e.g. because of health issues)
    - ☐ Clients forget appointments
    - ☐ Clients are afraid of the costs

	True	False	Don't know
1. Losing teeth is part of ageing			
2. Medication use may cause oral health problems			
3. Brushing once a day is sufficient for older people			
4. People with full dentures should wear these during the night			
5. Clients with full dentures only need to see a dental professional, when they have complaints			
6. Soft or fluid foods are best for older people			
7. Uncleaned dentures look bad, but this does no harm to the oral health			
8. Loss of weight and oral health could be related			
9. Bad oral hygiene may contribute to the cause of fungal infections in the mouth			
10. A bad oral health can contribute to a declined quality of life			
11. A bad oral health may lead to dysregulation of blood sugar levels in diabetes patients			
12. In clients with a dry mouth, dental cavities and gum problems do occur less often			
13. A soft tooth brush is better, than a rigid brush, to brush the teeth of clients with natural teeth			
14. When clients eat or drink sweet foods more often than 7 times a day, their teeth are more likely to get cavities			
15. When the gums start bleeding when brushed softly, it is better to quite brushing			
16. When a home care nurse supports with, or performs oral care in clients, he or she should wear gloves			
17. Wearing full dentures has no consequences on the bacterial flora in the mouth of the client			
18. Rinsing the mouth with water, is a good alternative for teeth brushing			
19. If dentures are worn during the night, the mucosa underneath may get inflamed			
20. A dry mouth is often caused by medication use			
21. Dentures should not be cleaned with tooth paste			
22. Dental costs should always be paid by the client or the additional insurance			
23. A bad oral health can cause pneumonia			
24. Cavities and gum infections are always caused by bacteria			

25. If people have a bad breath, this is often caused by stomach or lung problems			
26. Products to clean dentures (detergent tablets) cleanse dentures, without further brushing needed			
27. When clients only receive tube feeding, oral care is not necessary anymore			
28. Dental implants should be left without further care (e.g. do not brush)			
29. The use of tooth paste with fluoride in it, is important for older people with natural teeth			

File S2 – OHAT and oral (self) care questionnaire (in Dutch and English)

Sûne Mûle

Naam en geslacht cliënt *Dhr* ◯ *Mw* ◯

………………………………………………………………

Geboortedatum cliënt ………………………………………………………………………………

Wat voor gebit heeft de cliënt? *Meerdere antwoorden mogelijk*

◯ natuurlijk gebit boven ◯ natuurlijk gebit onder ◯ prothese boven ◯ prothese onder

◯ anders……

Bezoekt de cliënt een tandarts? ◯ Ja ◯ Nee

Indien ja, wanneer was het laatste bezoek aan de tandarts?

aantal maanden geleden ◯ 0-6 ◯ 6-12 ◯ 12-24 ◯ anders nl ……………………..

Bezoekt de cliënt een mondhygiënist? ◯ Ja ◯ Nee

Indien ja, wanneer was het laatste bezoek aan de mondhygiënist?

aantal maanden geleden ◯ 0-6 ◯ 6-12 ◯ 12-24 ◯ anders nl ……………………..

Wie voert de mondzorg uit? *Meerdere antwoorden mogelijk*

◯ cliënt ◯ mantelzorger ◯ thuiszorgmedewerker ◯ anders nl ……………………..

Welke mondzorgactiviteiten worden uitgevoerd en hoe vaak ?

Tandenpoetsen	◯ 2x daags	◯ 1x daags	◯ om de dag	◯ anders nl ……………………..
Ragen	◯ 2x daags	◯ 1x daags	◯ om de dag	◯ anders nl ………………………..
Tandenstoken	◯ 2x daags	◯ 1x daags	◯ om de dag	◯ anders nl ………………………..

Prothese schoonmaken ◯ 2x daags ◯ 1x daags ◯ anders nl ……………………………………

Waarmee wordt de prothese schoongemaakt?

◯ tandpasta ◯ schoonmaakmiddel ◯ azijn ◯ anders nl ……………………………………………

Hoe wordt de prothese weggezet?

◯ in water ◯ in prothesebakje ◯ anders nl ……………………………………………………

Figure S1 OHAT Dutch

Categorie	0= gezond	1= verandering	2= ongezond	Score
**Lippen**	Glad<br>Roze<br>Vochtig	Droog<br>Schraal<br>Gebarsten of rode mondhoeken	Zwelling of bult op lip<br>Wit/rode/zwerende plek<br>Bloedende en/of zwerende mondhoeken	
**Tong**	Normaal<br>Vochtig<br>Roze<br>Ruw	Vlekkerig<br>Kloofjes aanwezig<br>Rood<br>Beslagen	Rode en/of witte plek<br>Zwerend en/ of gezwollen tong	
**Tandvlees + slijmvliezen**	Roze<br>Vochtig<br>Geen bloeding<br>Glad	Droog<br>Glanzend, ruw, rood, Gezwollen<br>Zweer onder prothetische voorziening	Gezwollen, gemakkelijk bloedend<br>Zweer<br>Wit en/ of rode plekken, algehele roodheid slijmvlies onder prothetische voorziening	
**Speeksel**	Vochtige slijmvliezen, Waterig en vrij vloeiend speeksel	Droge, kleverige slijmvliezen<br>Kleine hoeveelheid speeksel<br>Cliënt ervaart een droge mond	Slijmvliezen uitgedroogd en rood<br>Weinig tot geen speeksel<br>Dik speeksel<br>Cliënt ervaart een droge mond	
**Natuurlijke tanden en kiezen (ja/nee)**	Geen cariës<br>Geen afgebroken tanden of kiezen<br>Geen wortelresten	1 - 3 carieuze of afgebroken tanden/ kiezen/ wortelresten<br>Slijtage van de tanden/ kiezen	Meer dan 4 carieuze of afgebroken tanden/ kiezen/ wortelresten<br>Ernstige slijtage van het gebit<br>Minder dan 4 tanden/ kiezen aanwezig in de mond	
**Prothetische voorziening (ja/nee)**	De prothetische voorziening is helemaal heel<br>Wordt regelmatig gedragen	1 deel van de prothetische voorziening of een tand/kies gebroken<br>Prothetische voorziening wordt slechts 1-2 uur per dag gedragen<br>Loszittende prothetische voorziening	2 of meer delen van de prothetische voorziening of tanden/kiezen van de voorziening zijn gebroken<br>Prothetische voorziening ontbreekt<br>Prothetische voorziening wordt niet gedragen<br>Prothetische voorziening zit los en plakmiddelen zijn nodig	
**Mondhygiëne**	Schoon<br>Geen etensresten, plaque of tandsteen in de mond of onder/op prothetische voorziening	Etensresten, tandsteen, plaque in 1-2 gebieden van de mond of op/ onder een klein deel van de prothetische voorziening<br>Slechte adem	Etensresten, tandsteen, plaque in groot deel van de mond of op de prothetische voorziening<br>Erg slechte adem	
**Dentale pijn**	Geen gedragsmatige, verbale of fysieke signalen voor pijn in de mond	Signalen van pijn in mond:<br>- verbaal<br>- gedragsmatig, zoals aan het gezicht zitten, op de lippen bijten, niet eten, agressie	Signalen van pijn in mond:<br>- verbaal<br>- gedragsmatig, zoals aan het gezicht zitten, op de lippen bijten, niet eten, agressie<br>Én ook fysieke aanwijzingen van pijn (zwelling van wang of tandvlees, gebroken tanden of zweren)	
○ Doorverwijzen naar een tandarts of mondhygiënist bij invullen van score 1 of 2 bij 1 van de items<br>○ Cliënt of verzorger/familie weigert tandheelkundige behandeling of doorverwijzing<br>○ Vul een MondzorgPlan in en start mondhygiëne behandeling<br>○ Retour voor evaluatie van de mondgezondheid op ..-..-....				**Totaal score:**<br><br>**/16**

Sûne Mûle

Name and sex of client: sir ○ madam ○ ……………………………………….

Date of birth ……………………………………………………………………………………

What is the dental status of the client? Multiple answers possible.
○ natural dentition upper jaw ○ natural dentition lower jaw
○ full prosthesis upper jaw ○ full prosthesis lower jaw

Does the client visit a dentist? ○ Yes ○ No
If yes, when was the last visit to the dentist?
How many months ago? ○ 0-6 ○ 6-12 ○ 12-24 ○ ……….

Does the client visit a dental hygienist? ○ Yes ○ No
If yes, when was the last visit to the dental hygienist?
How many months ago? ○ 0-6 ○ 6-12 ○ 12-24 ○ ……….

Who performs daily oral care? Multiple answers possible.
○ client ○ informal care taker ○ home care nurse ○ different ……………………..

What oral care is performed and how often ?

Teeth brushing	○ 2 times per day	○ once per day	○ every other day	○ ……………..
Interdental brushes	○ 2 times per day	○ once per day	○ every other day	○ ……………..
Tooth picks	○ 2 times per day	○ once per day	○ every other day	○ ……………..

Denture cleaning ○ 2 times per day ○ once per day ○……………………………

What is used for denture cleaning?
○ tooth paste ○ cleansing agent ○ vinegar ○………………

How are dentures stored overnight?

○ in water ○ in denture case ○ ……………………..

Figure S2 Original OHAT – (Chalmers, 2004)

Resident:______________ Completed by:______________				Date:___/___/___
	Scores – *You can circle individual words as well as giving a score in each category (* if 1 or 2 scored for any category please organize for a dentist to examine the resident)*			
Category	0 = healthy	1 = changes*	2 = unhealthy*	Category scores
Lips	smooth, pink, moist	dry, chapped, or red at corners	swelling or lump, white/red/ulcerated patch; bleeding/ulcerated at corners	
Tongue	normal, moist roughness, pink	patchy, fissured, red, coated	patch that is red and/or white, ulcerated, swollen	
Gums and tissues	pink, moist, smooth, no bleeding	dry, shiny, rough, red, swollen, one ulcer/sore spot under dentures	swollen, bleeding, ulcers, white/red patches, generalized redness under dentures	
Saliva	moist tissues, watery and free flowing saliva	dry, sticky tissues, little saliva present, resident thinks they have a dry mouth	tissues parched and red, very little/no saliva present, saliva is thick, resident thinks they have a dry mouth	
Natural teeth Yes/No	no decayed or broken teeth/roots	1-3 decayed or broken teeth/ roots or very worn down teeth	4 + decayed or broken teeth/roots, or very worn down teeth, or less than 4 teeth	
Dentures Yes/No	no broken areas or teeth, dentures regularly worn, and named	1 broken area/tooth or dentures only worn for 1-2 hrs daily, or dentures not named, or loose	more than 1 broken area/tooth, denture missing or not worn, loose and needs denture adhesive, or not named	
Oral cleanliness	clean and no food particles or tartar in mouth or dentures	food particles/tartar/plaque in 1-2 areas of the mouth or on small area of dentures or halitosis (bad breath)	food particles/tartar/plaque in most areas of the mouth or on most of dentures or severe halitosis (bad breath)	
Dental pain	no behavioural, verbal, or physical signs of dental pain	are verbal &/or behavioural signs of pain such as pulling at face, chewing lips, not eating, aggression	are physical pain signs (swelling of cheek or gum, broken teeth, ulcers), as well as verbal &/or behavioural signs (pulling at face, not eating, aggression)	
□ Organize for resident to have a dental examination by a dentist<br>□ Resident and/or family/guardian refuses dental treatment<br>□ Complete Oral Hygiene Care Plan and start oral hygiene care interventions for resident<br>□ Review this resident's oral health again on Date:___/___/___				TOTAL SCORE: ___ 16

Oral Health Assessment Tool (OHAT) for Dental Screening (modified from Kayser-Jones et al (1995) by Chalmers (2004)).

File S3 – Results of the completed MIDI questionnaires by HCN at t0 and t1, determinants in 4 domains

Table S1 Results of the completed MIDI questionnaires by HCN at t0 and t1, determinants in 4 domains

T0 baseline (%)<br><br>T1 after 6 months (%)	Completely disagree/ completely not/ (almost) none of my colleagues/ don't know OCP	Disagree/ not/ a minority of my colleagues/ I know about OCP but not the content	nor agree, nor disagree / maybe not, maybe it does/ half of my colleagues/	Agree/ yes/ almost all my colleagues/ I know about OCP and some content	Completely agree/ completely yes/ all my colleagues/ I know all about OCP
Determinants concerning OCP, questions 1- 7					
1. OCP expectations are clear	1%<br>3%	9%<br>11%	39%<br>16%	50%<br>64%	1%<br>6%
2. OCP is based on true knowledge	1%<br>1%	7%<br>9%	40%<br>10%	50%<br>76%	2%<br>4%
3. OCP provides all information and materials for success	0%<br>2%	6%<br>9%	40%<br>15%	51%<br>64%	3%<br>10%
4. OCP is too difficult for me *	11%<br>25%	51%<br>62%	36%<br>10%	2%<br>3%	0%<br>0%
5. OCP fits in my usual way of working*	1%<br>0%	7%<br>7%	53%<br>30%	39%<br>57%	0%<br>6%
6. OCP effects are clearly visible to me	1%<br>1%	6%<br>13%	67%<br>51%	26%<br>31%	0%<br>4%
7. OCP is suitable for my clients*	0%<br>0%	6%<br>7%	42%<br>30%	49%<br>55%	3%<br>8%
Determinants concerning HCNs and older people, questions 8 – 18					
8. Clients become aware of the importance of oral care by OCP	0%<br>0%	6%<br>8%	31%<br>28%	60%<br>56%	3%<br>8%

9. OCP is time consuming for HCNs*	2%<br>3%	22%<br>50%	63%<br>36%	13%<br>10%	0%<br>1%
10. It is important to improve frail older people's oral health	1%<br>0%	3%<br>3%	9%<br>6%	69%<br>75%	18%<br>16%
11. I expect from OCP to actually improve my clients oral health	0%<br>0%	1%<br>0%	45%<br>51%	51%<br>47%	3%<br>3%
12. It is part of my job to perform oral care or to support clients	0%<br>0%	4%<br>4%	12%<br>6%	67%<br>63%	17%<br>27%
13. It is part of my job to observe client's oral (health) care	0%<br>0%	3%<br>4%	15%<br>6%	64%<br>67%	18%<br>23%
14. Clients will be content about OCP	0%<br>0%	3%<br>5%	56%<br>29%	39%<br>61%	2%<br>5%
15. Clients will participate in OCP	1%<br>0%	11%<br>14%	54%<br>53%	33%<br>33%	1%<br>1%
16. I can rely on help of my colleagues in OCP if needed	1%<br>1%	1%<br>2%	21%<br>14%	70%<br>75%	17%<br>8%
17. How large is the proportion of colleagues that will actually participate in OCP?*	1%<br>26%	5%<br>20%	15%<br>30%	62%<br>12%	17%<br>12%
18a. How much is expected from you to participate in OCP – by the health insurance company?	3%<br>2%	6%<br>3%	45%<br>47%	38%<br>38%	8%<br>10%
18b. How much is expected from	0%<br>0%	0%<br>0%	6%<br>7%	66%<br>58%	28%<br>35%

you to participate in OCP – by the dental hygienists of OCP?					
18c. How much is expected from you to participate in OCP – by your manager?	0%<br>0%	2%<br>1%	19%<br>21%	62%<br>63%	17%<br>15%
18d. How much is expected from you to participate in OCP – by the organization?	0%<br>0%	1%<br>1%	17%<br>17%	67%<br>66%	15%<br>15%
18e. How much is expected from you to participate in OCP – your colleagues/ team members?	0%<br>0%	0%<br>1%	15%<br>28%	71%<br>60%	14%<br>11%
Determinants concerning the organization, questions 19 – 28					
19a. If it comes to working with OCP, how much do you care about the opinion of – the health insurance company?	3%<br>7%	11%<br>16%	56%<br>46%	28%<br>27%	2%<br>4%
19b. If it comes to working with OCP, how much do you care about the opinion of – the dental hygienists of OCP?	0%<br>0%	0%<br>3%	26%<br>16%	66%<br>67%	8%<br>14%
19c. If it comes to working with OCP, how much do you care about the opinion of – the manager? *	0%<br>1%	2%<br>4%	40%<br>34%	54%<br>55%	4%<br>6%
19d. If it comes to working with OCP, how much do you care	0%<br>1%	1%<br>3%	38%<br>26%	55%<br>62%	6%<br>8%

about the opinion of – the organization?					
19e. If it comes to working with OCP, how much do you care about the opinion of – your colleagues/ team members?	0%<br>0%	1%<br>1%	19%<br>21%	72%<br>69%	8%<br>9%
20. If you want to, do you think you can perform (or support) clients' oral care? *	1%<br>0%	0%<br>3%	34%<br>23%	57%<br>67%	8%<br>7%
21. If you want to, do you think you can assess clients' oral care/ status?*	0%<br>0%	0%<br>1%	34%<br>29%	62%<br>64%	4%<br>6%
22. How much do you know about the content of the OCP? *	29%<br>1%	32%<br>2%	-	35%<br>45%	4%<br>52%
23. Are there agreements within your team/ organization about participating in OCP (in work plans or in work policy, etcetera?) *	19% (no)<br>14%	-	(I don't know)<br>45%<br>67%	-	14% (yes)<br>45%
24. Our organization enables me with enough time to fit OCP in my daily work *	(no)<br>6%<br>11%	-	(neutral)<br>68%<br>38%	-	(yes)<br>26%<br>51%
25. In my team one or more persons are designated to coordinate the	12% (no)<br>9%	-	(I don't know)<br>59%<br>33%	-	(yes)<br>29%<br>58%

implementation of OCP					
26. Are there other organizational changes expected in the near future? (reorganization/ merges/ staff turnover etcetera)	(no)<br>23%<br>39%	-	(I don't know)<br>43%<br>24%	-	(yes)<br>34%<br>37%
27. In my team I have easy access to information and materials of the OCP	1%<br>0%	2%<br>4%	73%<br>24%	23%<br>66%	1%<br>6%
28. There are regular feedback moments about the progress of implementation of OCP in my team*	3%<br>1%	8%<br>18%	74%<br>39%	13%<br>38%	2%<br>4%
Determinants concerning the social and political environment, question 29					
29. OCP matches with current laws, rules and guidelines/ procedures *	1%<br>1%	2%<br>0%	60%<br>32%	33%<br>65%	4%<br>2%

* % are significantly different from baseline, α<0.05

File S4 – Squire Checklist
Research and reporting methodology
Revised Standards for QUality Improvement Reporting Excellence (SQUIRE 2.0)

**Text section and item name**	**Page**
	**info is located**
**Title and abstract**	**1**
1. **Title**	1
Indicate that the manuscript concerns an initiative to improve healthcare (broadly defined to include the quality, safety, effectiveness, patient-centredness, timeliness, cost, efficiency and equity of healthcare).	1
2. **Abstract**	1
a. Provide adequate information to aid in searching and indexing.	1
b. Summarise all key information from various sections of the text using the abstract format of the intended publication or a structured summary such as: background, local problem, methods, interventions, results, conclusions.	1
**Introduction: Why did you start?**	**2**
3. **Problem description** - Nature and significance of the local problem.	
4. **Available knowledge** - Summary of what is currently known about the problem, including relevant previous studies.	2-3
5. **Rationale** - Informal or formal frameworks, models, concepts and/or theories used to explain the problem, any reasons or assumptions that were used to develop the intervention(s) and reasons why the intervention(s) was expected to work	2-3
6. **Specific aims** - Purpose of the project and of this report.	4
**Methods: What did you do?**	4
7. **Context** - Contextual elements considered important at the outset of introducing the intervention(s).	4-5
8. **Intervention(s)**	4-5
a. Description of the intervention(s) in sufficient detail that others could reproduce it.	4-5
b. Specifics of the team involved in the work.	4-5
9. **Study of the intervention(s)**	5-6
a. Approach chosen for assessing the impact of the intervention(s).	5-6

b. Approach used to establish whether the observed outcomes were due to the intervention(s).	5-6
10. **Measures**	5-6
a. Measures chosen for studying processes and outcomes of the intervention(s), including rationale for choosing them, their operational definitions and their validity and reliability.	5--7
b. Description of the approach to the ongoing assessment of contextual elements that contributed to the success, failure, efficiency and cost.	5-7
c. Methods employed for assessing completeness and accuracy of data.	6-7
11. **Analysis**	8
a. Qualitative and quantitative methods used to draw inferences from the data.	8
b. Methods for understanding variation within the data, including the effects of time as a variable.	8
12. **Ethical considerations** - Ethical aspects of implementing and studying the intervention(s) and how they were addressed, including, but not limited to, formal ethics review and potential conflict(s) of interest.	5
**Results: What did you find?**	
13. **Results**	9-12
a. Initial steps of the intervention(s) and their evolution over time (eg, time-line diagram, flow chart or table), including modifications made to the intervention during the project.	9-12
b. Details of the process measures and outcomes.	9-12
c. Contextual elements that interacted with the intervention(s).	9-12
d. Observed associations between outcomes, interventions and relevant contextual elements.	9-12
e. Unintended consequences such as unexpected benefits, problems, failures or costs associated with the intervention(s).	9-12
f. Details about missing data.	9-12
**Discussion: What does it mean?**	
14. **Summary**	13
a. Key findings, including relevance to the rationale and specific aims.	13-14
b. Particular strengths of the project.	13-14
15. **Interpretation**	13-16
a. Nature of the association between the intervention(s) and the outcomes.	13-16
b. Comparison of results with findings from other publications.	13-16
c. Impact of the project on people and systems.	13-16
d. Reasons for any differences between observed and anticipated outcomes, including the influence of context.	13-16

e. Costs and strategic trade-offs, including opportunity costs.	13-16
16. **Limitations**	14
a. Limits to the generalisability of the work.	14
b. Factors that might have limited internal validity such as confounding, bias or imprecision in the design, methods, measurement or analysis.	14
c. Efforts made to minimise and adjust for limitations.	14
**Conclusions**	16
a. Usefulness of the work.	16
b. Sustainability.	16
c. Potential for spread to other contexts.	16
d. Implications for practice and for further study in the field.	16
e. Suggested next steps.	16
**Other information**	In title page / acknowledgements
18. **Funding** - Sources of funding that supported this work. Role, if any, of the funding organisation in the design, implementation, interpretation and reporting.	

Ogrinc G, et al. BMJ Qual Saf 2015;0:1–7. doi:10.1136/bmjqs-2015-004411
Downloaded from http://qualitysafety.bmj.com/ on January 2, 2017
